# Effects of lipid supplementation on milk fatty acid profile of lactating cows under semi-confined summer conditions in southern Brazil

**DOI:** 10.1007/s11250-026-05147-x

**Published:** 2026-06-15

**Authors:** Ione Maria Pereira Haygert-Velho, Andressa Faccenda, Diego do Prado Vargas, Júlia Laize Bandeira Calgaro Lorini, Caroline Veiga Elicker, José Laerte Nörnberg, Dileta Regina Moro Alessio, Marcos Busanello, João Pedro Velho

**Affiliations:** 1 Departamento de Zootecnia e Ciências Biológicas, Universidade Federal de Santa Maria, Palmeira das Missões, Palmeira das Missões, Brazil; 2 Instituto de Saúde e Produção Animal, Universidade Federal Rural da Amazônia., Belém, Brazil; 3 Professor do curso de Medicina Veterinária, e do Programa de Pós-graduação em Tecnologia Ambiental, Universidade de Santa Cruz do Sul., Santa Cruz do Sul, Brazil; 4 Zootecnista e Mestre em Agronegócios, Ijuí, Brazil; 5 Departamento de Tecnologia e Ciência dos Alimentos, Universidade Federal de Santa Maria, Santa Maria, Brazil; 6 Centro Universitário Leonardo Da Vinci, Indaial, Brazil; 7 Departamento de Zootecnia, Universidad Federal do Paraná, Curitiba, Brazil

**Keywords:** Dairy cows, Lipid supplementation, Heat stress, Milk fatty acids, Summer conditions

## Abstract

Heat stress is a major challenge in tropical and subtropical dairy systems; however, limited evidence exists on how dietary lipid supplementation influences cow behavior, physiology, and milk quality under semi-confined summer conditions. This study evaluated the effects of different lipid sources (protected fat, flaxseed, and a mixture) on milk yield, composition, fatty acid profile, and behavioral and physiological responses of Holstein cows. Twelve cows were allocated to three 4 × 4 Latin squares, with each period lasting 15 days (10 days adaptation, 5 days data collection). Treatments consisted of a basal diet supplemented with 278 g protected fat, 790 g flaxseed, or a combination of 139 g protected fat and 395 g flaxseed, compared with a control. Behavioral observations, physiological parameters, and milk samples were collected. Results showed that daily thermal load indicated moderate thermal challenge, but behavioral and physiological responses remained within normal ranges. Milk yield and protein content were unaffected by lipid supplementation. Flaxseed significantly increased milk fat percentage and improved the fatty acid profile, with higher concentrations of unsaturated fatty acids, including omega-3 and CLA, compared with protected fat. Somatic cell count did not differ among treatments, indicating no adverse effect on udder health. Elevated milk urea nitrogen values suggested imbalances between protein and carbohydrate supply. Overall, flaxseed supplementation enhanced milk nutritional quality while maintaining production stability under moderate heat stress. These findings suggest that flaxseed may represent a practical nutritional strategy to improve milk value and support sustainable dairy management in warm climates.

## Introduction

The ongoing rise in global temperatures has extended the effects of heat stress beyond subtropical regions into temperate climates (Polsky and von Keyserlingk 2017). Heat stress affects animals across all production levels and is recognized as an important environmental challenge to modern dairy systems. Extended daylight, elevated temperatures, high relative humidity (RH), and inadequate shading in summer result in critical thermal conditions that adversely affect dry matter (DM) intake, thereby diminishing milk yield and composition (Fagan et al. 2010; Chen et al. 2024). Recent studies published in *Tropical Animal Health and Production* (TAHP) have reinforced that high temperature–humidity index (THI) values are consistently linked to physiological alterations, reduced feed intake, and decreased milk performance and quality parameters in grazing and semi-confined dairy cows (de Lima Guimarães Yamada et al. 2023; Gheno et al. 2024).

Dairy cattle production has made substantial progress over the past century, primarily due to genetic improvement programs, especially in countries such as the United States, Canada, Germany, and Israel. The selection objectives for dairy cows have progressively shifted from an exclusive focus on milk yield to a more comprehensive approach that encompasses fertility, health, longevity, temperament, welfare, and environmental sustainability, including greenhouse gas mitigation (Miglior et al. 2017). Recent studies indicate that productive performance is increasingly influenced by environmental gradients and genotype–environment interactions, especially under challenging climatic conditions. This underscores the need for integrative management and nutritional strategies (Martins et al. 2025).

Heat stress adversely affects dairy cow welfare and productivity; however, early prediction and management of thermal risk can partially mitigate these negative effects. Nevertheless, numerous mitigation efforts remain insufficient to maintain adequate homeothermic balance. Heat stress is typically recognized by observable clinical symptoms alongside increased ambient temperature and RH, as well as physiological indicators, such as rectal temperature, respiratory rate, and heart rate (Herbut et al. 2018). Research in tropical and subtropical production systems indicates that even moderate increases in THI may alter behavioral patterns, increase metabolic load, and compromise milk yield and composition (de Lima Guimarães Yamada et al. 2023; Gheno et al. 2024).

Nutritional strategies have emerged as effective tools to mitigate the detrimental impacts of thermal stress. Among these, the inclusion of dietary lipids has garnered interest for its ability to enhance dietary energy density without proportionally increasing metabolic heat production. The incorporation of fatty acids into the diet of dairy cows can be highly beneficial, particularly for their effects on fertility and reproductive efficiency, as well as for improving the fatty acid composition of milk (Bionaz et al. 2020). Over the past decade, interest in the milk lipid fraction has intensified due to its recognized nutraceutical potential and relevance to human health (Bernard et al. 2018).

The scientific community increasingly recognizes that diets supplemented with unsaturated fats can help reduce greenhouse gas emissions from ruminants, especially methane (CH₄) (Beauchemin et al. 2020). Flaxseed has been widely investigated as a lipid source because of its high α-linolenic acid (C18:3 n-3) content, which has been shown to effectively inhibit ruminal methanogenesis and alter milk fatty acid profiles in dairy cows (Benchaar et al. 2015). Recent experimental evidence confirms that flaxseed or linseed oil supplementation enhances the concentration of beneficial polyunsaturated fatty acids, particularly omega-3, while either maintaining or slightly modifying productive performance, contingent on inclusion level and dietary composition (Koçoğlu et al. 2025; Gheno et al. 2024). Furthermore, improvements in productive efficiency are recognized as a key factor in methane mitigation strategies (Roques et al. 2024).

The inclusion of vegetable oils and oilseeds in ruminant diets correlates with improvements in the nutritional quality of milk fat for human consumption, notably through increased conjugated linoleic acid (CLA) and omega-3 fatty acids, which are associated with cardioprotective and anti-inflammatory benefits (Oliveira et al. 2021). In addition, studies evaluating alternative nutritional and environmental interventions have demonstrated that dietary manipulation may interact with climatic conditions to influence physiological parameters, milk stability, and fatty acid composition (Gheno et al. 2024). Complementary research has also shown that climatic variability affects udder health and milk quality, reinforcing the complex interaction between environment, nutrition, and productivity in dairy systems (Corrêa et al. 2024).

Lipid supplementation in dairy cow diets represents a multifactorial strategy that can affect feed intake, milk yield, milk composition, and milk fatty acid profile, all of which are important for animal performance and human health. In this context, and given the growing importance of sustainable and climate-resilient dairy production systems, this study aimed to evaluate the effects of incorporating various lipid sources into the diets of semi-confined Holstein cows during summer on milk production, milk composition, milk fatty acid profile, and animal behavior.

We hypothesized that flaxseed supplementation would improve the milk fatty acid profile and increase the concentration of beneficial unsaturated fatty acids without negatively affecting milk yield, physiological responses, or behavioral patterns of dairy cows under semi-confined summer conditions.

The findings of this study are anticipated to improve the understanding of how dietary lipid supplementation affects milk composition, fatty acid profile, and animal responses under practical semi-confined summer conditions. Because cows were maintained under a semi-confined grazing system, total dry matter intake from pasture could not be accurately quantified. Therefore, the effects of lipid supplementation on productive and metabolic responses should be interpreted cautiously, particularly regarding nutrient intake and energy balance.

## Materials and methods

The experiment, developed by the research group Inovazoot (Management in the Integration of Crop and Ruminant Production Systems), was conducted as a partnership between the Celeste Gobbato State Technical School (EETCG) and Federal University of Santa Maria – Palmeira das Missões Campus from January to March 2011. Note that the historical climatic data from this 2011 experimental window directly represent the semi-confined summer stress conditions evaluated herein. The regional climate was classified as humid subtropical with hot summers (Cfa) according to Alvares et al. (2013), while the soil was classified as a typical dystrophic Red Latosol (LVd3) (Streck et al. 2002).

Twelve Holstein cows (black and white), homogeneous in age, lactation stage, lactation number, and milk yield, were used. Animals were allocated to three 4 × 4 Latin squares, with two consisting of multiparous cows and one comprising primiparous cows. Each experimental period spanned 15 days, with the first 10 days used for animal adaptation and the subsequent five days for data collection on DM intake, milk production, animal behavior, and physiological parameters.

Cows were assigned to the following treatments: Control (C), Protected Fat (PF), Flaxseed (FL), and Mixture (M). All treatments were administered a uniform basal diet consisting of forage sorghum pasture (*Sorghum bicolor*), Tifton pasture (*Cynodon* sp.), oat silage (*Avena* sp.), and a conventional concentrate composed of ground corn, soybean meal, wheat bran, and mineral supplement (Table 1). The treatments differed solely in lipid supplementation: 278 g of protected fat in PF, 790 g of whole flaxseed in FL, and 139 g of protected fat plus 395 g of whole flaxseed in M.

**Table 1:** Composition of the concentrate used in all treatments

Ingredient	Percentage
Corn grain	42.2
Soybean meal (46% CP)	35.0
Wheat bran	17.4
Mineral-vitamin premix*	4.4
Common salt	1.0

*Mineral–vitamin premix composition per kg of product: calcium 190 g; phosphorus 78.8 g; sulfur 39.2 g; potassium 9.8 g; magnesium 19.6 g; cobalt 120 mg; copper 735 mg; iodine 39.2 mg; zinc 2,450 mg; manganese 1,636 mg; selenium 19.6 mg; sodium 85 g; fluorine (max.) 800 mg; antioxidant 2,475 mg; phosphorus solubility in 2% citric acid = 90%; vitamin A 205,800 IU; vitamin D₃ 58,800 IU; vitamin E 784 mg

The sorghum and Tifton pastures were subdivided into strips using electric fencing. The strip size was established based on DM availability per hectare, with the objective of providing 10 kg of DM per 100 kg of body weight. An intermittent grazing system was adopted, with one strip used per day and a rest period determined by forage growth rates. Pasture DM availability was assessed by harvesting four samples of 0.25 m² per strip prior to animal entry and measuring residual forage post-animal exit. In sorghum pasture, DM density was measured at 30-cm intervals along plant height owing to the heterogeneity of this species, which may affect voluntary intake.

Following the morning milking (05:30 h), cows were moved to sorghum paddocks and returned at 11:00 h to headlocks, where they were administered 50% of their daily oat silage and concentrate allocation with the respective treatments. At 13:30 h, animals were transferred to a shaded paddock with unrestricted access to water, where they remained until the afternoon milking (16:00 h). After milking, they received the remaining 50% of oat silage, concentrate, and the corresponding lipid source. At 19:00 h, cows were moved to Tifton paddocks, where they remained until the subsequent morning milking.

Silage, concentrate, and lipid source intakes were measured individually by weighing the amounts offered and refusals, recorded in both the morning and afternoon.

On the 11th and 12th days of each experimental period, milk samples were collected from each cow. Composite samples were formed proportionally from morning and afternoon milk yields. Samples for determining fat, protein, lactose, total solids, somatic cell count, and milk urea nitrogen were collected in sterile containers containing bronopol preservative and dispatched to the Dairy Herd Analysis Service (SARLE) of the University of Passo Fundo (UPF), Passo Fundo, RS, Brazil, within 24 h. Another portion of the composite sample was stored in amber bottles and frozen at -20 °C for fatty acid profile analysis.

On the 13th day of each experimental period, animal behavior was evaluated for 24 h. Animals were observed every 10 min to record grazing, idle, and rumination times. Simultaneously, environmental data were collected every 2 h, encompassing ambient temperature (digital thermometer), RH (thermo-hygrometer), and wind speed (propeller anemometer). At the same intervals, the external body surface temperature of the cows was measured using an infrared thermometer at several anatomical points, including the jaw, chest, and mammary gland. At three points during the day (06:00, 12:00, and 16:00 h), rectal temperature was measured using a mercury thermometer inserted into the rectal ampulla for 3 min. Heart and respiratory rates were recorded by counting heartbeats through the caudal artery and observing flank movements for 20 s, respectively. The values were multiplied by three to obtain beats or movements per minute. On the 14th day of each experimental period, body condition score was estimated according to NRC (2001), and body weight was measured using a weight tape.

The THI was calculated using the equation proposed by Baccari et al. (1983, cited by Souza et al. 2004):

1 $${\mathbf{THI}}={\mathbf{T_{s}}}+{\mathbf{0.36}}{\mathbf{Td}}+{\mathbf{41.2}}$$

where Ts is dry-bulb temperature (°C), and Td is dew point temperature (°C). THI values were interpreted according to Azevedo et al. (2005), in which values ≤ 70 indicated normal environmental conditions, 70–72 denoted an alert situation, and 72–78 exceeded the critical threshold for milk production, causing production decline.

Milk yield was corrected to 3.5% fat using the formula proposed by Tyrrell and Reid (1965, cited by Leiva et al. 2000):

2 $${\mathbf{FCM}}={(\textbf{12.82}} \times {\mathbf{Ffat})}+ {({\mathbf{7.13}}\times{\mathbf{Fprotein}})}+{({\mathbf{0.323}}\times{\mathbf{MY}})}$$

where MY is milk yield (kg day⁻¹), Ffat is fat yield (kg day⁻¹), and Fprotein is protein yield (kg day⁻¹).

The milk fatty acid profile was determined after lipid extraction, followed by saponification and esterification of fatty acids. This was conducted via gas chromatography (Agilent 6890) with a fused-silica capillary column SP-2560 (100 m, recommended for CLA determination) and flame ionization detector (FID). Chromatographic runs lasted approximately 70 min. Injector and detector temperatures were 250 °C and 300 °C, respectively. Injection was performed in split mode at a 21:1 ratio. Hydrogen served as the carrier gas, with a flow rate of 40 mL min⁻¹ and head pressure of 18 psi. Fatty acid peaks were identified through comparisons with standard retention times (CLA mixture containing cis-9, trans-11 and cis-10, trans-12 octadecadienoic acid esters – Sigma; vaccenic acid methyl ester – Sigma; and a 37-component fatty acid methyl ester mix – Supelco). Nonadecanoic acid was used as the internal standard. Results were expressed as g per 100 g of total fatty acids.

Data were analyzed using ANOVA appropriate for a replicated 4 × 4 Latin square design using Minitab software (McKenzie and Goldman 1999). The statistical model included the fixed effects of treatment and period, while square and cow nested within square were considered random effects. The model can be represented as:

$$\:{Y}_{ijkl}=\mu\:+{T}_{i}+{P}_{j}+{S}_{k}+{C}_{\left(S\right)l}+{e}_{ijkl}$$

where $\:{Y}_{ijkl}$ is the dependent variable, $\:\mu\:$ is the overall mean, $\:{T}_{i}$ is the fixed effect of treatment, $\:{P}_{j}$ is the fixed effect of period, $\:{S}_{k}$ is the random effect of square, $\:{C}_{\left(S\right)l}$ is the random effect of the cow nested within the square, and $\:{e}_{ijkl}$ is the residual error.

Physiological variables measured repeatedly throughout the day were analyzed as repeated measures over time, including treatment, time, and treatment × time interaction effects. The covariance structure that best fitted the data was selected according to Akaike’s Information Criterion. The individual cow was considered the experimental unit. Means were compared using Tukey’s test, and significance was declared at *P* < 0.05.

## Results and discussion

**Fig. 1:**
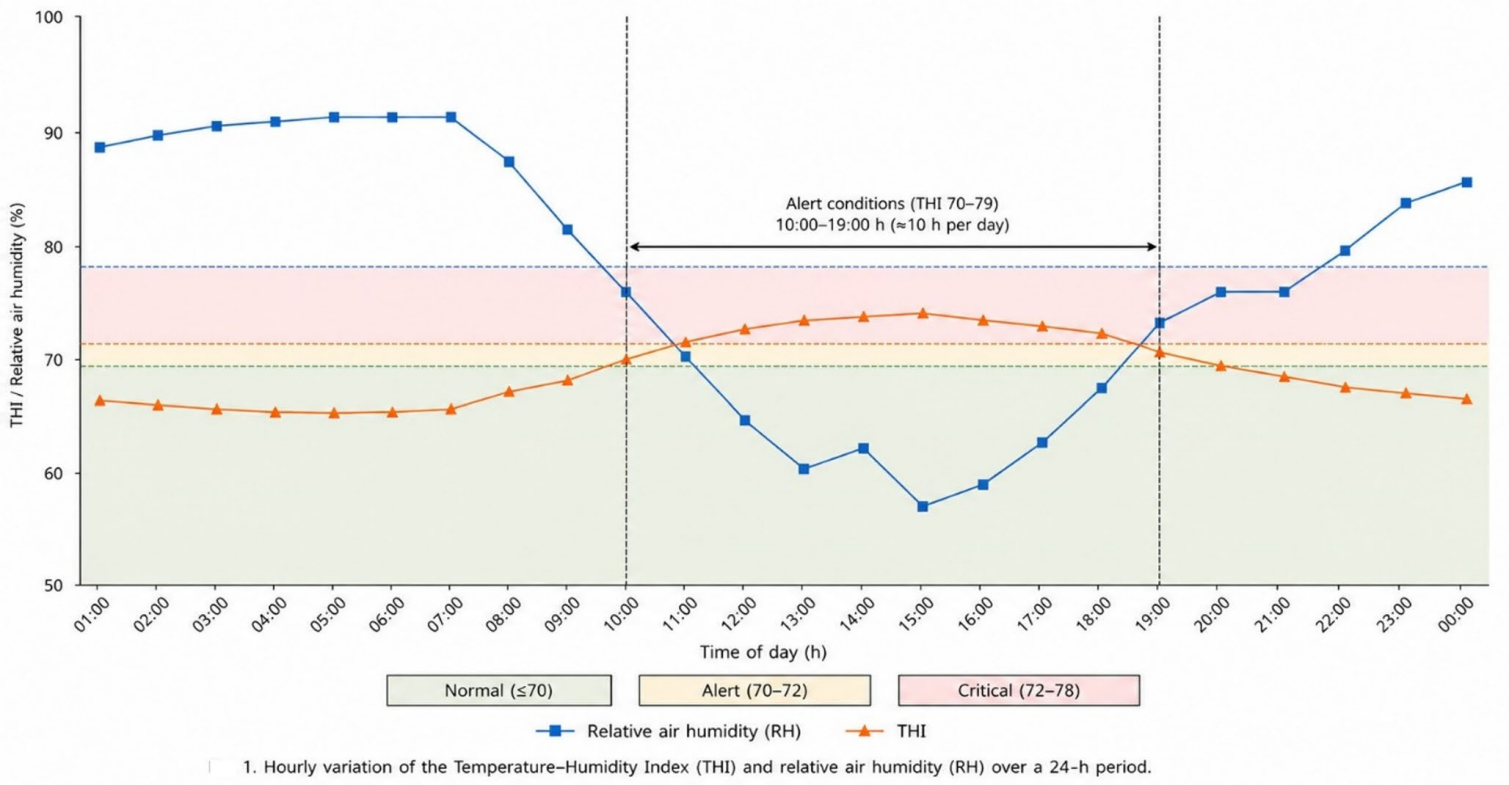
Hourly variation of the temperature-humidity index (THI) and relative air humidity (RH) over a 24 h period

**Fig. 2:**
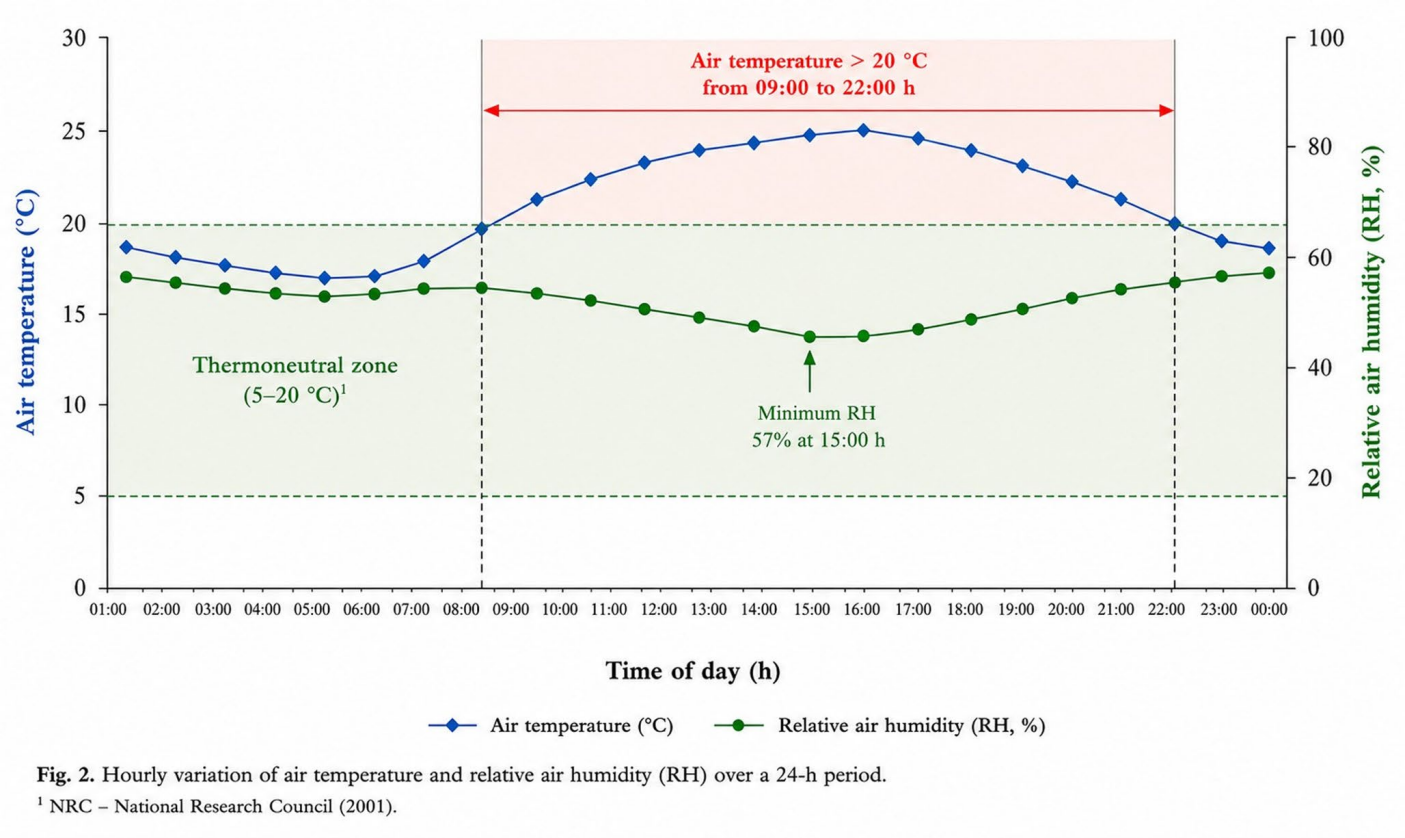
Hourly variation of air temperature and relative air humidity (RH) over a 24 h period

Monitoring RH and ambient temperature during the experimental period facilitated assessment of their effects on the responses of lactating cows to heat stress. At night, RH was higher than that during the day (Fig. 1). However, temperatures were milder (Fig. 2), remaining within the thermoneutral zone (5–20 °C) as defined by NRC (2001). In contrast, daytime RH values were lower, reaching a minimum of 57% at 15:00 h, while air temperature from 09:00 h to 22:00 h surpassed the thermoneutral zone.

The THI integrates the combined effects of air temperature and RH on the animal. Therefore, daytime interactions among these environmental variables suggest that the cows experienced heat stress for approximately 10 h per day. As per the previously described THI variation, values observed between 10:00 h and 19:00 h characterize an alert condition regarding the influence of environmental factors on the animals (70–79).

The duration and proportion of the main behavioral activities performed by the cows during the 24-h observation period are presented in Table 2. Many of these activities are closely associated with feed intake. Behavioral plasticity serves as a key adaptive mechanism in response to environmental challenges. Vizzotto et al. (2015) and Reis et al. (2021) reported that even under moderate thermal stress, access to shade alters the physiological and behavioral attributes of grazing cows, leading to changes in both social and ingestive behaviors.

**Table 2:** Behavior of holstein cows supplemented with different lipid sources

Variables	*N*	Treatment					SEM	*P*-value
		Control	Fat	Flaxseed	Mixture			
Idle time (h)	32	7.23	7.26	7.57	7.55	0.366		0.6135
Idle time (%)	32	30.6	30.9	32.0	31.8	1.54		0.6624
Rumination time (h)	32	8.97	9.03	8.66	8.72	0.353		0.6145
Rumination time (%)	32	37.8	38.1	36.6	36.8	1.48		0.6081
Grazing time (h)	32	3.89	3.63	3.74	3.71	0.378		0.7121
Grazing time (%)	32	16.4	15.4	15.8	15.7	1.59		0.7681
Feed bunk time (h)	32	2.67	2.50	2.68	2.48	0.170		0.3987
Feed bunk time (%)	32	11.3	10.5	11.4	10.4	0.72		0.3492
Walking time (h)	32	0.88	0.98	0.89	1.10	0.214		0.5982
Walking time (%)	32	3.57	4.12	3.76	4.64	0.900		0.5449
Water intake time (h)	23	0.23	0.24	0.24	0.24	0.050		0.9890
Water intake time (%)	23	0.90	0.85	1.03	0.86	0.255		0.9079
Feed bunk DM intake (kg)	48	11.2	11.0	11.0	10.8	0.37		0.1651
Feed bunk DM intake (% BW)	35	1.95	2.03	2.00	1.99	0.118		0.4951

Feed provided at the feeder constituted a considerable portion of the diet, with an average of 2 h and 35 min devoted to this activity (Table 2), corresponding to 40.9% of the total time spent on feed consumption. The peak feeder intake occurred between 17:00 and 18:00 h, consistent with the findings of Damasceno et al. (1999) on the behavior of Holstein cows with both constant and limited access to shade. Pollock et al. (2022) reported similar findings in both Holstein and Holstein × Jersey crossbred cows.

The C group exhibited a longer grazing time relative to that of the other treatments, which can be attributed to its lower dietary energy density. Across all treatments, grazing activity peaked after the morning milking, with a secondary peak after the afternoon milking, thereby confirming that animals preferentially graze during milder thermal windows.

Greater grazing activity after morning milking likely reflected cooler environmental conditions and differences in forage availability between periods.

In the 24-h evaluation of rumination time distribution, animals exhibited a higher frequency of rumination at night. Fraser (1980) reported that cattle exhibited increased rumination after sunset, which subsequently declined before sunrise as a new grazing period commenced. The same author emphasized that within a 24-h period, an animal could ruminate 15 to 20 times, with each rumination bout lasting between 2 min and 1 h.

Animals provided with shade generally exhibit longer rumination times under heat stress conditions (Reis et al. 2021), supporting the behavioral adaptations observed in the present study.

Among the activities performed by the animals, idle time represented, on average, 31% of the observed period. When treatments were compared, the C group exhibited shorter idle time, reflecting increased time allocated to grazing and rumination activities (Table 2). Although the peak of this activity was observed during the morning milking, the period between 11:00 h and 19:00 h accounted for 42.8% of the total idle time. This finding corroborates that of Damasceno et al. (1999) and Pereira et al. (2023), which indicated a higher frequency of idleness during periods of greater solar radiation. Notably, the animals remained in a paddock with limited shade availability between 13:00 and 16:00 h, potentially leading to increased idle time.

Cows were primarily observed engaging in locomotion activities when moving toward pastures and the milking parlor. The energy expenditure associated with these displacements is considerable, as certain paddocks were located on sloped terrain, necessitating greater physical effort from the animals. Regarding treatments, cows administered the M treatment spent the most time in locomotion, whereas those in the C treatment moved the least (Table 2). This observed difference may be attributed to lipid sources, as C animals exhibited reduced dietary selectivity due to the greater need for intake to meet their nutritional requirements. Locomotion activity was mainly associated with displacement to pastures and the milking parlor. Greater locomotion observed in lipid-supplemented treatments may reflect selective ingestive behavior rather than discomfort, as the nutritional energy density influences exploratory feeding patterns. Indirect evidence of comparable behavioral modulation due to lipid inclusion was reported in trials examining milk fatty acid enhancement during summer periods (Gheno et al. 2024).

Water consumption time remained below 1%, reinforcing evidence of restricted availability. Restricted water availability likely reduced drinking frequency and may have influenced thermoregulatory responses and productive performance. Adequate water access is essential under heat stress conditions, particularly for lactating dairy cows (Cardot et al. 2008; Ammer et al. 2018).

The evaluation of physiological parameters indicated that mean rectal temperature values, regardless of treatment, remained within the normal range (Table 3). This corroborates the findings of Martello et al. (2004), who reported values between 37.5 and 39.3 °C. However, the gradual increase in this variable throughout the day is associated with the circadian rhythm of body temperature, which typically reaches its maximum in the late afternoon and minimum during the early morning hours. Higher temperatures were noted in animals without access to shade (Kendall et al. 2006). A study by Daltro et al. (2017) in Minas Gerais involving Holstein cows reported a mean rectal temperature of 40.84 °C. Rectal temperature is the most widely used indicator of core body temperature and is frequently utilized as a physiological indicator of heat stress (Brito et al. 2020). Physiological parameters demonstrated that rectal temperature remained within the normal range, even with a circadian elevation observed in the afternoon. Similar stability of rectal temperature during moderate thermal load has been observed in dairy cows subjected to environmental or nutritional mitigation strategies, such as dietary lipid supplementation (de Lima Guimarães Yamada et al. 2023; Gheno et al. 2024). Heart rate increases at 12:00 and 16:00 h reflected transient heat load, a phenomenon also documented in controlled summer trials involving nutritional additives (Gheno et al. 2024).

**Table 3:** Mean values of physiological parameters in Holstein cows subjected to diets with different lipid sources

Variables	*N*	Treatment				SEM	*P*-value
		Control	Fat	Flaxseed	Mixture		
Rectal Temperature (6:00 h) (°C)	32	38.1	38.1	38.1	38.1	0.19	0.9543
Rectal Temperature (12:00 h) (°C)	32	38.8	39.0	38.9	38.8	0.21	0.3922
Rectal Temperature (16:00 h) (°C)	32	39.1	39.0	39.1	38.9	0.31	0.6828
Heart Rate (6:00 h) (beats/min)	32	67.1	72.2	64.9	69.3	2.85	0.3007
Heart Rate (12:00 h) (beats/min)	32	73.9	78.1	75.2	74.3	3.02	0.3464
Heart Rate (16:00 h) (beats/min)	32	73.8	74.6	72.8	71.4	4.14	0.7799
Respiratory Rate (6:00 h) (breaths/min)	32	45.2	54.3	54.0	50.0	3.47	0.1008
Respiratory Rate (12:00 h) (breaths/min)	32	58.0	61.9	58.1	55.2	5.84	0.6215
Respiratory Rate (16:00 h) (breaths/min)	32	68.0	69.4	68.6	62.3	8.81	0.6235

Physiological heart rate values did not differ among treatments (*P* > 0.05) (Table 3). According to Reece (1996), the normal heart rate of cattle ranges from 60 to 70 beats min⁻¹. Therefore, at 06:00 h, the PF treatment exceeded this limit, while the remaining treatments remained within the normal range. At 12:00 and 16:00 h, an increase in heart rate was noted across all treatments, with all values exceeding 70 beats min⁻¹, indicating increased thermal challenge to the animals during these periods. Notably, although altered heart rate values were observed at 12:00 and 16:00 h, the M treatment presented lower values at these times compared with those of the other treatments.

In extreme heat conditions, thermoregulatory mechanisms are activated, leading to an elevated heart rate and subsequent increase in body temperature, alongside a decrease in DM intake to minimize total thermal load (de Andrade Ferrazza et al. 2017). Mean values close to 99 beats min⁻¹ were observed in evaluations carried out in Minas Gerais, Brazil, where the average daily temperature ranged from 20.7 to 37.9 °C and RH reached 95% (Daltro et al. 2017).

The respiratory rate recorded at 06:00 h did not indicate stress in any treatment, as values below 60 breaths min⁻¹ were considered non-stressful, those between 60 and 80 breaths min⁻¹ were classified as alert indicators, and those above 80 breaths min⁻¹ indicated dangerous conditions for the animals (Pacheco et al. 2020). At 12:00 h, stress was observed in the FL treatment, as the mean respiratory rate exceeded the physiologically adequate threshold. At 16:00 h, stress was evident across all treatments, with the M treatment exhibiting the lowest effect (Tables 2 and 3). Respiratory rate changes can result from several factors, including body size, age, exercise level, excitement, ambient temperature, pregnancy, digestive tract fill degree, and health status (Reece 1996). The respiratory rate followed a similar pattern, with alert or stress values during warmer periods. This respiratory modulation aligns with adaptive thermoregulatory responses reported in semi-confined and pasture-based dairy systems subjected to summer heat stress (Koçoğlu et al. 2025).

Milk yield and fat-corrected milk yield at 3.5% fat (Table 4) did not differ among treatments (*P* > 0.05). In terms of milk fat production, the FL treatment presented the highest value, differing significantly from that of the PF treatment (*P* < 0.05), both in terms of percentage and kg cow⁻¹ day⁻¹. The inclusion of a mixture of extruded soybean and flaxseed grains (100 g kg⁻¹) in the diet of lactating cows has been shown to increase milk yield and non-fat solids despite a decrease in milk fat percentage; nevertheless, improvements in milk fat quality have also been reported (Dawod et al. 2020).

**Table 4:** Mean values of milk yield, milk composition, and somatic cell count in Holstein cows subjected to a diet supplemented with different lipid sources

Variables	*N*	Treatment				SEM	*P*-value
		Control	Fat	Flaxseed	Mixture		
Milk Yield (kg/day)	48	24.1	25.3	24.4	24.5	0.88	0.1864
Fat-Corrected Milk (3.5%) (kg/day)	48	23.0	23.0	23.7	22.8	0.89	0.2908
Total Milk Solids (%)	48	11.48ab	11.24b	11.65a	11.32b	0.177	0.0042
Total Milk Solids (kg/day)	48	2.81 ab	2.75 b	2.85 a	2.77 b	0.043	0.0031
Fat (%)	48	3.35 ab	3.04 b	3.49 a	3.19 ab	0.132	0.0115
Fat (kg/day)	48	0.82 ab	0.74 b	0.85 a	0.78 ab	0.031	0.0082
Lactose (%)	48	4.47	4.50	4.44	4.46	0.056	0.5831
Lactose (kg/day)	48	1.10	1.10	1.09	1.10	0.014	0.6308
Protein (%)	48	2.82	2.82	2.87	2.83	0.049	0.2358
Protein (kg/day)	48	0.69	0.69	0.70	0.69	0.012	0.2489
Milk Urea Nitrogen (mg/dL)	36	17.1	16.2	17.9	16.9	1.08	0.0738
Somatic Cell Count (cells/mL × 1,000)	46	58.9 ± 23.29	69.0 ± 28.66	106.3 ± 41.77	108.7 ± 44.34		0.3825

Total solids production, expressed both as percentage and as kg cow⁻¹ day⁻¹, differed significantly among treatments (*P* < 0.05). The PF and M treatments exhibited the lowest total solids content, likely due to their lower milk fat concentration. Total solids differed across treatments primarily due to variations in fat percentage. Controlled dietary trials assessing flaxseed inclusion levels have similarly indicated that lipid supplementation can alter milk fat concentration while maintaining overall milk yield (Koçoğlu et al. 2025).

The total solids percentage for the PF treatment was significantly different from that of the other treatments (*P* < 0.05). Oliveira et al. (2021) reported a decrease in total solids percentage in milk from cows that were fed flaxseed oil, likely indicating a decrease in fat yield. Because total solids correspond to the sum of fat, protein, and lactose, reduced fat production directly affects the total solids percentage.

Oliveira et al. (2021) reported a decrease in both the percentage and yield of milk fat in animals fed a diet containing 2.5% flaxseed oil. In their study, the forage-to-concentrate ratio was 50:50, and the neutral detergent fiber (NDF) level might have contributed to this reduction. Petit (2010) demonstrated that whole flaxseed supplementation had limited effects on milk fat and protein contents, while Huang et al. (2021) reported no significant differences in milk yield.

The lower milk fat content observed in cows administered diets containing protected fat may be attributed to the dissociation of calcium salts at a low ruminal pH. This occurs because the lipid source is provided alongside the concentrate, whose fermentation reduces ruminal pH approximately 2 h after ingestion (Nocek et al. 2002). Under these conditions, protected fat may lose its inert characteristics in the rumen and become susceptible to ruminal biohydrogenation.

Lipid sources rich in polyunsaturated fatty acids, such as protected fat, can modify the lipid profile of milk fat by increasing CLA; therefore, supplementation with oilseeds in the diets of lactating cows may positively affect desirable milk components for human consumption (Plata-Pérez et al. 2022). According to NRC (2001), CLA isomers or their metabolites with a trans double bond at position 10 of the carbon chain inhibit milk fat synthesis.

The trans-10, cis-12 isomer inhibits the lipogenic activity of specific enzymes, leading to reduced *de novo* synthesis and consequent milk fat depression. Notably, flaxseed serves as a source of polyunsaturated fatty acids; therefore, a reduction in milk fat content was expected with its inclusion. However, the FL treatment resulted in higher milk fat values. A possible explanation is that the oil contained within the flaxseed grain may have experienced more complete ruminal biohydrogenation, likely reducing the formation of the trans-10, cis-12 isomer and thereby contributing to the maintenance of milk fat levels.

Lactose production, expressed as both a percentage and in kg cow⁻¹ day⁻¹, did not differ significantly among the examined treatments (*P* > 0.05). This aligns with the findings of Côrtes et al. (2010), who investigated the inclusion of whole flaxseed and calcium salts of flaxseed oil and similarly found no differences in lactose production. Protein production, measured as both percentage and kg cow⁻¹ day⁻¹, did not significantly differ among treatments (*P* > 0.05), with results similar to those reported by Vilela et al. (2002) and Santos et al. (2001).

Milk urea nitrogen values were relatively high, indicating an imbalance between protein and carbohydrate supply. This imbalance may be attributed to the low carbohydrate content of the diet and linked to the provision of low-quality oat silage.

Somatic cell count did not differ significantly among treatments (*P* > 0.05). Oliveira et al. (2021) observed similar results, indicating that somatic cell count was not significantly affected by the inclusion of soybean and flaxseed oils. Therefore, the potential immunological benefits of flaxseed lignans were not distinctly evident. Somatic cell count did not differ across treatments, suggesting no detrimental effect on udder health. Climatic influences on milk quality and mastitis incidence have been strongly highlighted in regional TAHP analyses, indicating that environmental stressors frequently outweigh dietary lipid sources in determining somatic cell dynamics (Corrêa et al. 2024).

Table 5 presents the profiles of short-, medium-, and long-chain fatty acids in milk for each treatment group. No significant effect was observed (*P* = 0.3025) for butyric acid (C4:0), a characteristic milk fatty acid important in *de novo* synthesis. Likewise, no significant effect (*P* > 0.05) was observed for caproic (C6:0) and caprylic (C8:0) acids. Fatty acids grouped as C10, including capric (C10:0) and caproleic (C10:1 n-1) acids, differed (*P* = 0.0243) between the C and M treatments, while showing no significant difference from the PF and FL treatments, respectively. Hendecanoic acid (C11:0) exhibited a similar pattern (*P* > 0.05). Lauric acid (C12:0) did not vary among treatments (*P* = 0.1700). Bergamo (2013) reported considerable reductions of approximately 15% in short- and medium-chain fatty acids (C4:0 to C16:0) following the inclusion of flaxseed in the diet.

**Table 5:** Milk fatty acid profile of holstein cows subjected to diets supplemented with different lipid sources during summer

Variables	*N*		Treatment						SEM		*P*-value
			Control	Fat		Flaxseed		Mixture			
C4	48	2.96		3.20	2.95		2.78		0.246	0.3025	
C6	48	1.35		1.35	1.38		1.13		0.092	0.0759	
C8	48	1.11		1.21	1.20		1.11		0.074	0.5259	
C10	48	3.33 a		3.21 ab	3.04 ab		2.83 b		0.189	0.0243	
C11	48	0.27 a		0.17 b	0.16 b		0.14 b		0.048	0.0013	
C12	48	1.66		1.58	1.55		1.46		0.097	0.1700	
C14	48	9.56 a		8.62 b	8.75 b		8.47 b		0.356	0.0022	
C14_1n5	48	0.61		0.60	0.61		0.60		0.038	0.9883	
C15	48	1.08 a		1.00 ab	0.99 ab		0.98 b		0.027	0.0414	
C16	48	30.7		28.6	29.2		28.9		0.74	0.0751	
C16_1n7	48	1.26		1.19	1.21		1.22		0.049	0.5643	
C17	48	0.7 a		0.62 b	0.65 ab		0.66 ab		0.023	0.0181	
C18	48	14.9 b		15.7 ab	16.5 a		16.5 a		0.59	0.0470	
C18:1 elaidic acid	48	0.53 b		0.57 ab	0.52 b		0.62 a		0.027	0.0062	
C18:1 vaccenic acid	48	2.02 b		2.86 a	1.91 b		2.58 a		0.160	< 0.0001	
C18_1n9C	48	25.1 b		26.5 ab	26.6 ab		26.9 a		0.69	0.0257	
C18_2n6C	48	1.69 ab		1.83 a	1.54 b		1.73 a		0.070	0.0014	
C20	48	0.28		0.28	0.27		0.27		0.012	0.8587	
C18_3n3	48	0.31 b		0.31 b	0.38 a		0.34 b		0.014	< 0.0001	
C18_2_c9_t11_CLA1	48	0.62 a		0.69 a	0.53 b		0.71 a		0.053	< 0.0001	
Saturated fatty acids	48	67.9 a		65.5 b	66.7 ab		65.3 b		0.73	0.0046	
Unsaturated fatty acids	48	32.1 a		34.5 b	33.3 ab		34.7 b		0.73	0.0046	

Myristic acid (C14:0) concentration was significantly higher (*P* = 0.0022) in the C treatment than in the lipid-supplemented diets. The fatty acid C14:1n5 did not vary (*P* = 0.9883) among lipid sources. Pentadecanoic acid (C15:0) differed based on dietary treatment (*P* = 0.0414), with the greatest amplitude observed between the C and M treatments. Palmitic (C16:0) and palmitoleic (C16:1n7) acids did not show significant variation among treatments. Margaric acid (C17:0) significantly differed (*P* = 0.0181) between the C and PF treatments, whereas no significant differences were observed (*P* > 0.05) when compared with the remaining diets.

The higher proportion of myristic acid in the C treatment aligns with the known effects regarding the impact of unsaturated lipid supplementation on reducing *de novo* synthesis of short- and medium-chain fatty acids in the mammary gland, as these fractions are primarily synthesized from acetate and β-hydroxybutyrate produced in the rumen (Lock and Bauman 2003; Shingfield et al. 2010). Recent nutritional trials confirm that incorporating oilseeds or protected fats reduces C14:0 and C16:0 concentrations, thereby improving the nutritional profile of milk fat for human health.

A higher proportion of stearic acid (C18:0) was noted in the FL and M treatments, which were not significantly different (*P* > 0.05) from the PF treatment; the latter treatment also showed no significant difference from the C treatment. Maia et al. (2006) assessed oil sources in goat diets and found that lipid supplementation led to increased stearic acid concentrations, with notable differences primarily between the C and supplemented diets. Stearic acid serves as a principal end-product of the ruminal biohydrogenation process of unsaturated fatty acids, with its increase frequently linked to the inclusion of polyunsaturated-fatty-acid-rich feeds (Jenkins et al. 2008; Shingfield et al. 2010).

Fatty acids with 18 carbon atoms containing one or two double bonds were significantly affected by treatments (*P* < 0.05). Elaidic acid (C18:1 trans-9) in the M treatment significantly differed (*P* = 0.0062) from that in the C and FL treatments but not from that in the PF treatment, which also showed no significant differences from the other treatments. The observed responses reflect modifications in ruminal biohydrogenation pathways and the relative abundance of trans-isomers, strongly affected by dietary lipid sources, forage-to-concentrate ratios, and ruminal pH stability (Bauman and Griinari 2003).

Petit (2015) assessed the impact of varying inclusion levels of flaxseed on mid-lactation cows, noting that most milk fatty acid proportions were altered by diet, while only a few fatty acids (4:0, cis-9 16:1, 17:0, and 22:5) remained unchanged. Although the milk fatty acid profile was nutritionally improved, incorporating whole flaxseed at inclusion levels exceeding 46 g/kg of DM decreased milk component yields. More recent meta-analyses corroborate that moderate inclusion of flaxseed or other oilseeds enhances unsaturated fatty acid content; however, excessive inclusion may inhibit milk fat synthesis and overall solids.

Regarding the fatty acid profile, lipid supplementation promoted expected shifts toward higher unsaturated fractions without severe depression of *de novo* synthesis.

This study demonstrates that the strategic inclusion of lipid sources in the diets of lactating Holstein cows managed under semi-confined conditions during summer can be adopted without detrimental effects on milk yield, basic milk composition, or key welfare indicators. Although behavioral and most physiological responses remained within normal biological ranges, the observed daily thermal load highlights the importance of environmental management. Specifically, shade availability, ventilation, and unrestricted access to high-quality water are essential components of dairy production systems in warm periods.

From a nutritional standpoint, flaxseed supplementation substantially enhanced the milk fatty acid profile by increasing the proportion of unsaturated and potentially health-promoting fatty acids, all while maintaining productive performance. Conversely, protected fat exhibited a neutral or intermediate effect, indicating that the selection of lipid source and its physical form plays a decisive role in modulating ruminal biohydrogenation and mammary lipid synthesis. These responses reinforce the concept that lipid supplementation should be aligned with both energetic supply and the intended qualitative characteristics of milk fat.

The absence of significant effects on somatic cell count and most physiological variables indicates that moderate lipid inclusion, when appropriately balanced within the diet, does not compromise animal health or metabolic stability. Nevertheless, the observed relatively elevated milk urea nitrogen values suggest that improved synchronization between protein and fermentable carbohydrate supply may further enhance nitrogen utilization efficiency and promote environmental sustainability.

## Limitations

Some limitations of the present study should be acknowledged. Because cows were maintained under grazing conditions, total pasture dry matter intake could not be accurately measured, limiting precise interpretation of nutrient intake and energetic responses. In addition, restricted water availability during part of the day likely influenced physiological and behavioral responses under heat stress conditions. Therefore, productive and metabolic responses should be interpreted cautiously.

## Conclusion

Flaxseed supplementation improved the milk fatty acid profile of semi-confined Holstein cows during summer by increasing the proportion of unsaturated fatty acids, including omega-3, without negatively affecting milk yield. Lipid supplementation did not substantially alter behavioral or physiological responses under the thermal conditions evaluated. However, because total pasture dry matter intake was not accurately quantified and water availability may have influenced animal responses, the productive and metabolic results should be interpreted cautiously. Further studies under more controlled intake and environmental conditions are necessary to confirm these responses under practical dairy conditions.

## Data Availability

Data will be made available on request.
